# In vitro inhibition of transmissible gastroenteritis coronavirus replication in swine testicular cells by short hairpin RNAs targeting the ORF 7 gene

**DOI:** 10.1186/1743-422X-9-176

**Published:** 2012-08-28

**Authors:** Lei He, Yan-ming Zhang, Ling-juan Dong, Min Cheng, Jing Wang, Qing-hai Tang, Gang Wang

**Affiliations:** 1 College of Veterinary Medicine, Northwest A & F University, Yangling, Shaanxi, 712100, China; 2 State Key Laboratory of Veterinary Biotechnology, Harbin Veterinary Research Institute, Chinese Academy of Agricultural Sciences, Harbin, Heilongjiang, 150001, China

**Keywords:** Transmissible gastroenteritis virus, RNA interference, Short hairpin RNA, ORF 7

## Abstract

**Background:**

Transmissible gastroenteritis (TGE) is a highly contagious viral disease of swine, characterized by severe vomiting, diarrhea, and high mortality. Currently, the vaccines for it are only partially effective and no specific drug is available for treatment of TGE virus (TGEV) infection. RNA interference has been confirmed as a new approach for controlling viral infections. In this study, the inhibitory effect of short hairpin RNAs (shRNAs) targeting the ORF 7 gene of TGEV on virus replication was examined.

**Results:**

Four theoretically effective sequences of TGEV ORF 7 gene were designed and selected for construction of shRNA expression plasmids. In the reporter assays, three of four shRNA expression plasmids were able to inhibit significantly the expression of ORF 7 gene and replication of TGEV, as shown by real-time quantitative RT-PCR analysis of viral ORF 7 and N genes and detection of virus titers (TCID_50_/ml). Stable swine testicular (ST) cells expressing the shRNAs were established. Observation of the cytopathic effect and apoptosis, as well as a cell proliferation assay demonstrated that the three shRNAs were capable of protecting ST cells against TGEV destruction, with high specificity and efficiency.

**Conclusions:**

Our results indicated that plasmid-transcribed shRNAs targeting the ORF 7 gene in the TGEV genome effectively inhibited expression of the viral target gene and viral replication in vitro. These findings provide evidence that the shRNAs have potential therapeutic application for treatment of TGE.

## Background

Transmissible gastroenteritis (TGE) is a highly contagious viral disease of swine, characterized by severe vomiting, diarrhea, and high mortality. These syndromes are especially serious in neonatal piglets aged <2 weeks old, frequently leading to a mortality as high as 100%; even surviving older pigs generally show growth retardation and low reward to feeding [1-3]. TGE causes enormous economic loss annually worldwide. Currently, several vaccines for prevention of TGE are available, but their efficacy is variable. Both the attenuated and inactivated vaccines are partially effective; attenuated TGE virus (TGEV) vaccines have the risk of reverting to a virulent form and may even induce an adverse reaction, and the inactivated ones are poorly protective in swine [4,5]. Moreover, newborn piglets infected with TGEV may die within 1–4 days, whereas the vaccines for TGEV do not provide effective protection at 7 days after administration [6]. However, no cure for TGE is presently available apart from symptomatic treatment. RNA interference (RNAi) has been confirmed as a post-transcriptional gene silencing mechanism, which is widely considered to be the major antiviral system in plants and insects [7,8]. The effective inhibition of replication of several RNA and DNA viruses in animal cells also has been reported by means of RNAi [9-13]. Therefore, RNAi may be developed as a potential therapy for TGE.

TGEV is a member of the genus *Alphacoronavirus* in the family *Coronaviridae*. It is a positive-sense, ssRNA virus with a 28.5-kb genome that contains a leader sequence at the 5′ end and a poly(A) tail at the 3′ end, and encodes four structural proteins [spike (S), envelope (E), membrane (M) and nucleoprotein (N)] and five nonstructural proteins (replicase 1a, 1b, 3a, 3b and protein 7) [14-16]. The genome itself, together with six sub-genomic mRNAs transcribed discontinuously, forms a nested set of RNAs of different lengths with co-terminal ends [17].

ORF 7 is located at the 3′ end of the genome. It encodes a 78-amino-acid, 9.1-kDa hydrophobic protein, and has been reported to play a role in the process of membrane-associated replication complexes and/or virion assembly [18,19]. Recently, more studies have shown that the deletion of TGEV ORF 7 by reverse genetics may be related to the viral virulent and promotes an intensified dsRNA-activated host antiviral response [14]. Compared to other viral proteins’ gene (such as proteins S, E, M or N), the ORF7 region is relatively conservative and RNAi targeting to ORF 7 gene will result in the degradation of both the sub-genomic mRNA for ORF 7 and the other sub-genomic mRNAs [18,20]. Besides, data showed that the 3' end of TGEV genome (ORF 7 gene and the downstream gene) could interact with host cell proteins and played a positive role in the replication of TGEV [21,22]. According to the characteristics of RNAi, the downstream gene will be degraded with the ORF 7 gene. Then the interaction of 3' end of TGEV genome and host cell proteins will be interrupted and the viral replication will be reduced. Thus, it is helpful to use RNAi against it as a new therapeutic option. Here, we demonstrate that RNAi targeting of the ORF 7 gene of TGEV, introduced by short hairpin RNAs (shRNAs), is capable of inhibiting virus replication and protecting swine testicular (ST) cells from the destruction induced by TGEV, which may be not only a new anti-TGEV strategy, but also a new approach to the study of its pathogenesis.

## Results

### Examination of shRNAs effect by real-time quantitative RT-PCR

**Figure 1:**
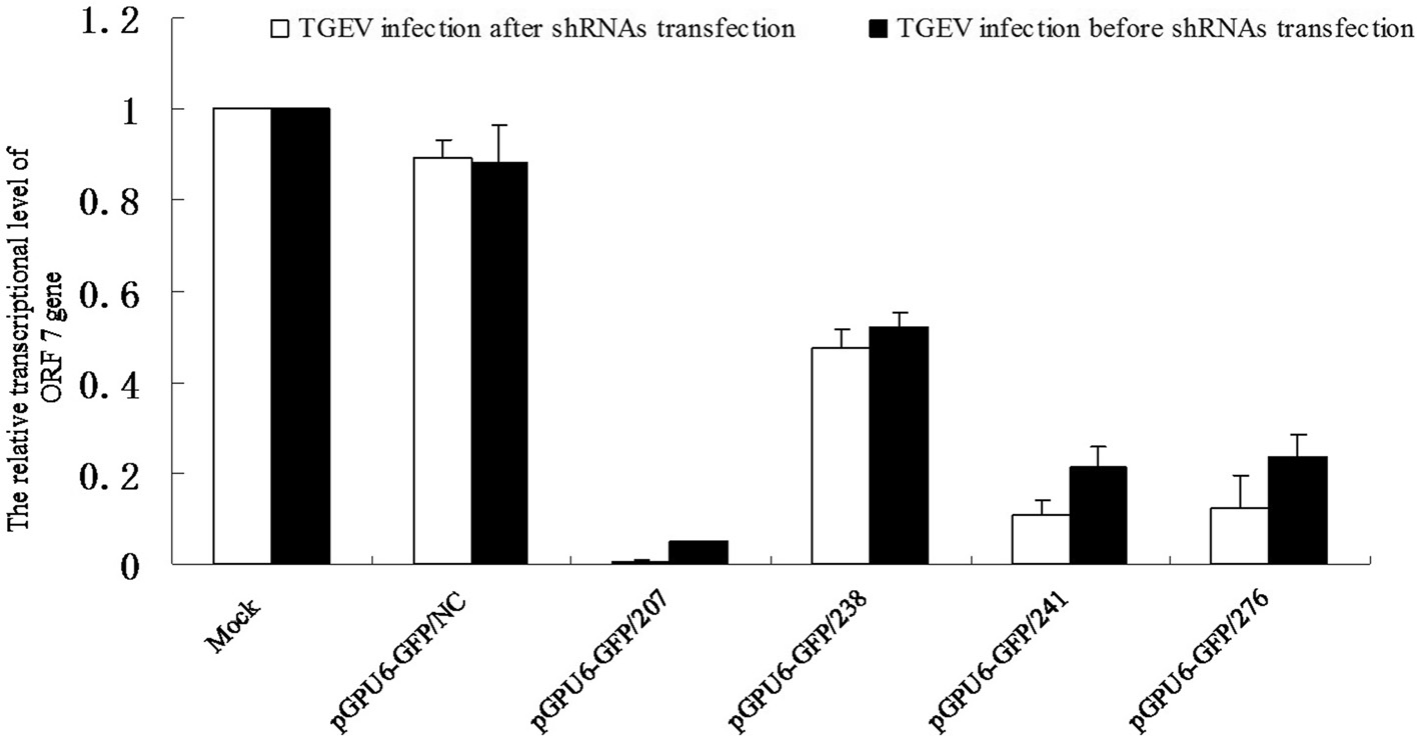
**Reduction in expression of TGEV ORF 7 mRNA levels by shRNAs directed against ORF 7 gene in ST cells.** Real time RT-PCR analysis of ORF 7 mRNA level was normalized to the corresponding β-actin in the same sample. The ORF 7 gene level in Mock controls was assigned a value of 1 for each experiment. The mean of three repeat experiments performed in triplicate is shown, and error bars represent the standard deviation (SD).

Relative quantifications of both the ORF 7 and N genes were performed by real-time quantitative RT-PCR. Comparative threshold (Ct) cycle values in three independent experiments were calculated by the Ct method, and the average relative amount of ORF 7 gene in each sample is represented in Figure 1. The relative amount of ORF 7 gene in mock control cells was regarded as 1.000, where the relative amounts of ORF 7 gene in cells infected with TGEV after being transfected with pGPU6-GFP/207, pGPU6-GFP/238, pGPU6-GFP/241, pGPU6-GFP/276 and pGPU6-GFP/NC were 0.006, 0.474, 0.108, 0.124 and 0.892, respectively, and in cells infected with TGEV before transfection was 0.050, 0.521, 0.212, 0.234 and 0.881, respectively. The sequence-specific shRNA pGPU6-GFP/207 reduced the amount of ORF 7 gene by approximately 99% and 95%, which was better than with the other three plasmids.

**Figure 2:**
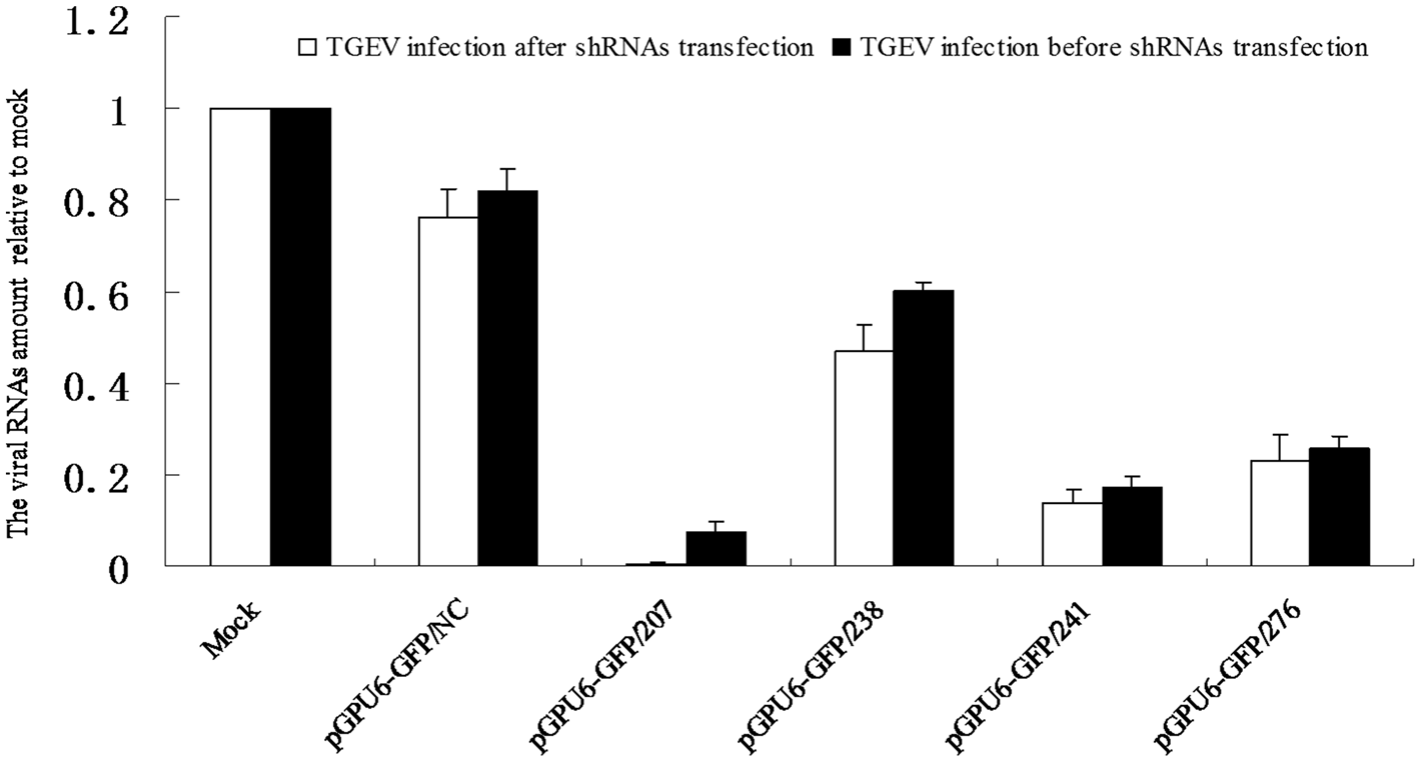
**Inhibition of TGEV RNA replication by shRNAs in ST cells.** The amount of viral genome (included sub-genomic mRNAs except the shortest one) was measured by real-time RT-PCR of the N gene, which was normalized to the corresponding β-actin in the same sample. The mean of three repeat experiments performed in triplicate is shown, and error bars represent the SD.

Real-time quantitative RT-PCR of the N gene (Figure 2) for cells infected with TGEV after transfection with those shRNAs was 0.006, 0.471, 0.138, 0.230 and 0.762, respectively. This indicated that these sequence-specific shRNAs had approximately 99%, 53%, 86%, 77% and 24% reductions in TGEV genome (included sub-genomic mRNAs, except the shortest one). Although the levels of viral genome in the cells infected with TGEV before being transfected with shRNAs were 0.075, 0.600, 0.174, 0.256 and 0.819, corresponding to a 92%, 40%, 83%, 74% and 18% reductions in TGEV RNA. Taken together, RNAi against ORF 7 gene showed a dramatic reduction in the TGEV viral.

### Examination of shRNAs effect by infectious virus assay

**Figure 3:**
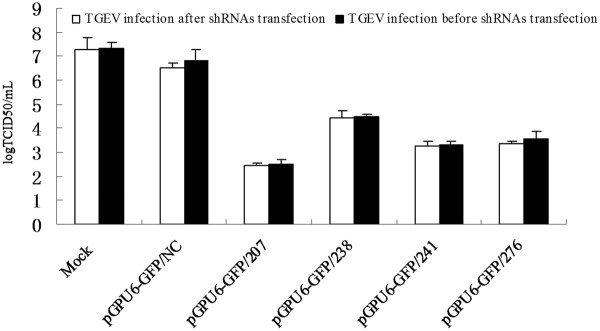
**Reduction in titers of TGEV in ST cells transfected with shRNA plasmids.** TCID_50_ values are the means of three repeat titrations, and error bars represent the SD.

The 50% tissue culture infective dose (TCID_50_) assay was performed to examine the effect of siRNA on production of viable virus. Figure 3 shows that the titers of TGEV were 10^2.51^, 10^4.48^, 10^3.31^, 10^3.54^ and 10^6.83^ TCID_50_/ml in cells infected with TGEV after being transfected with pGPU6-GFP/207, pGPU6-GFP/238, pGPU6-GFP/241, pGPU6-GFP/276 and pGPU6-GFP/NC at 48 h post-infection, respectively, and the titers of TGEV in cells infected with TGEV before being transfected with shRNAs were 10^2.43^, 10^4.40^, 10^3.25^, 10^3.35^ and 10^6.49^ TCID_50_/ml. These data indicated that RNAi against ORF 7 gene reduced the progeny virus production significantly; pGPU6-GFP/207 showed a maximum inhibition, whereas pGPU6-GFP/241 and pGPU6-GFP/276 showed partial virus replication inhibition, with pGPU6-GFP/238 being the least effective shRNA.

### Establishment of ST cell lines stably expressing shRNAs

**Figure 4:**
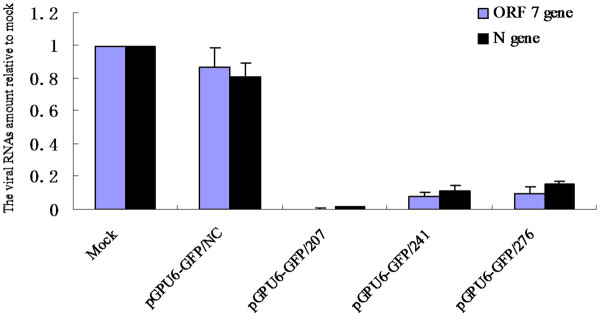
**Reduction in expression of TGEV ORF 7 and N gene mRNA levels in shRNAs expressing cells.** Real time RT-PCR analysis of ORF 7 and N gene mRNA level was normalized to the corresponding β-actin in the same sample. The mean of three repeat experiments performed in triplicate is shown, and error bars represent the SD.

**Figure 5:**
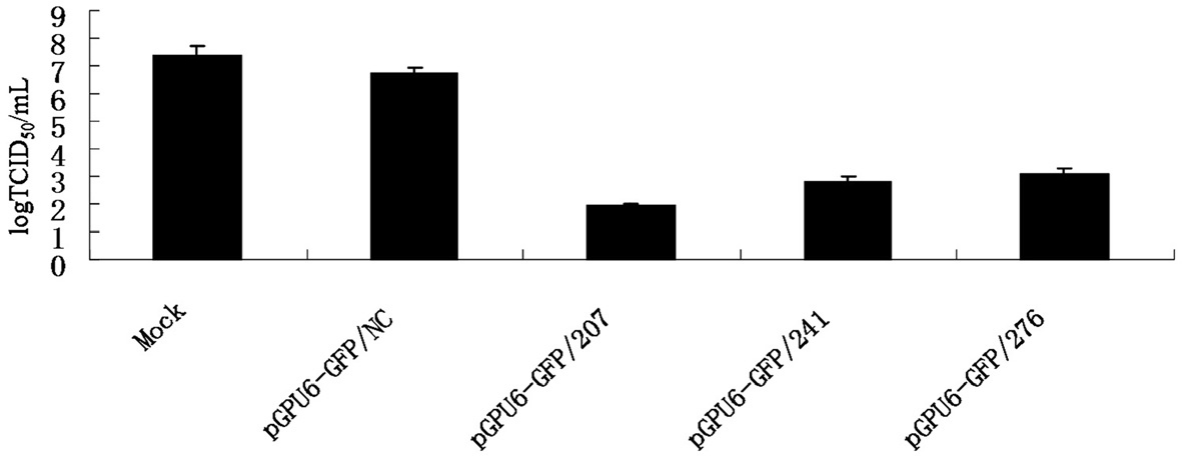
**Reduction in titers of TGEV in ST cells stably expressing shRNAs.** TCID_50_ values are the means of three repeat titrations, and error bars represent the SD.

Based on the results obtained by real-time quantitative RT-PCR and determination of TCID_50_, cells stably expressing pGPU6-GFP/207, pGPU6-GFP/241, pGPU6-GFP/276 and pGPU6-GFP/NC were selected by using G418 (pGPU6-GFP/238 abandoned). Green fluorescence protein (GFP) was used as a reporter and cells were screened until 90% of them showed green fluorescence. As shown in Figures 4 and 5, a similar virus replication inhibition trend was observed in the cell lines in comparison with the transiently transfected cells. The three shRNA-expressing cells exhibited potent ability in silencing TGEV RNAs, whereas cells expressing pGPU6-GFP/NC showed a slightly nonspecific effect.

### Examination of shRNA effect by cell proliferation assay

**Figure 6:**
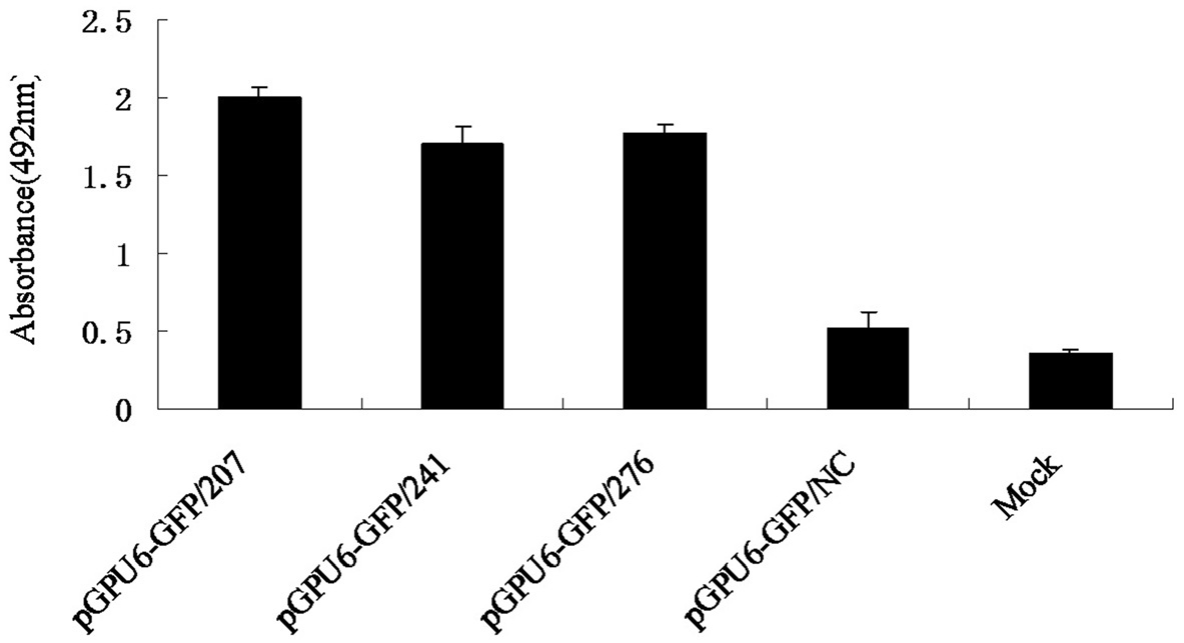
**shRNA effect on protection of ST cells by MTS assay.** The MTS assay was used to evaluate the viable cell numbers of each ST cell line stably expressing the shRNA. OD values represent the mean ± SD of three separate experiments performed six times.

To study the protective effect of shRNAs against TGEV destruction, the MTS cell proliferation assay was performed on ST cells stably expressing shRNA plasmids. As the absorbance is proportional to the number of living cells in culture, it was measured at 492 nm by using an ELISA reader at 40 h post-infection (including 4 h incubation with MTS). Mean OD_492_ of solutions in ST cells stably expressing pGPU6-GFP/NC and in control ST cells was 0.455 and 0.332, respectively, whereas in ST cells expressing pGPU6-GFP/207, pGPU6-GFP/241 or pGPU6-GFP/276 were 2.001, 1.707 and 1.772, respectively (Figure 6). This showed that ST cells expressing shRNAs, especially pGPU6-GFP/207, were protected from TGEV destruction, leading to a significant increase in living cells compared with cells stably expressing pGPU6-GFP/NC, or ST control cells.

### Examination of shRNA effect by fluorescence microscopy

**Figure 7:**
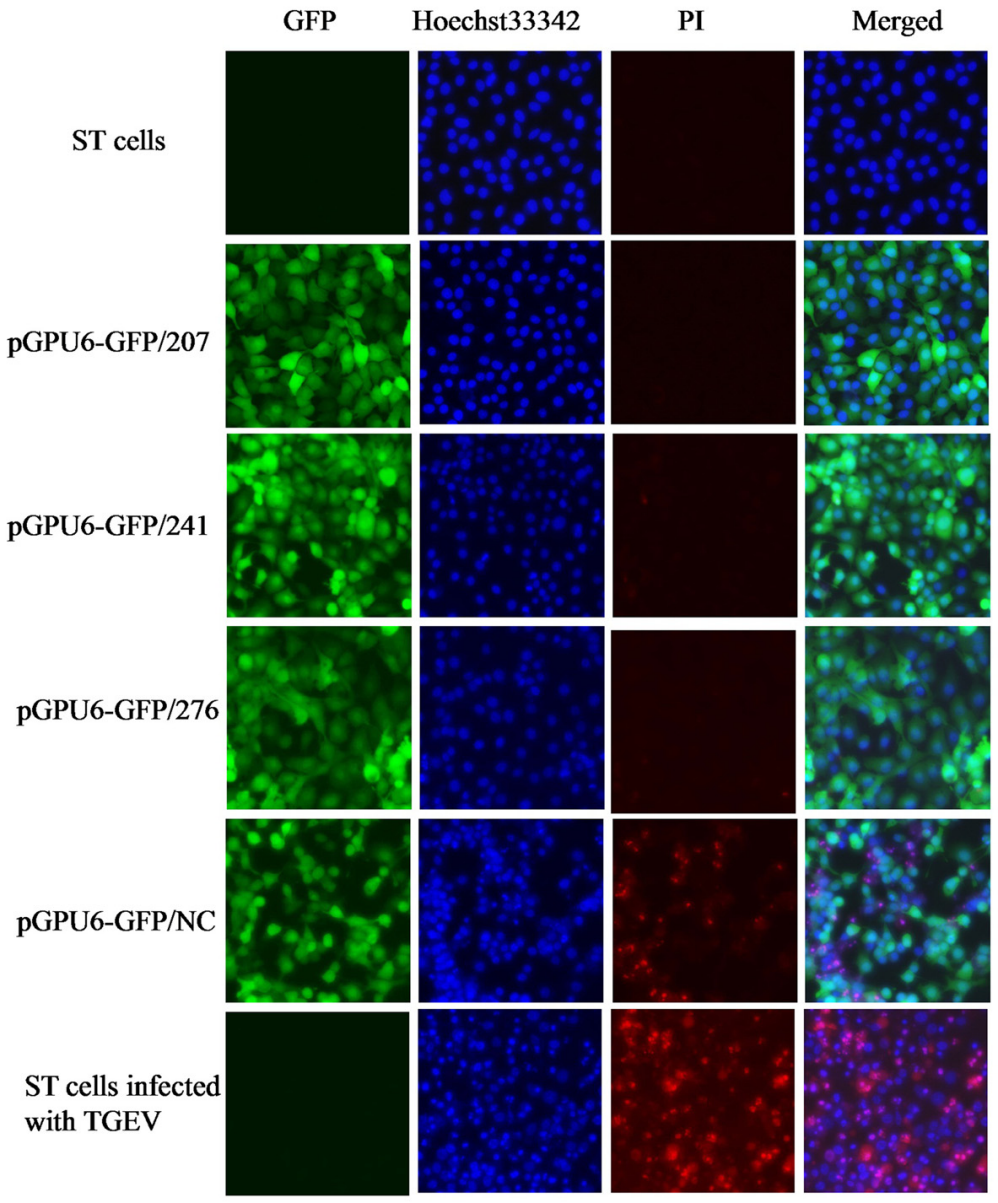
**Effect of shRNAs on protecting ST cells against TGEV-induced destruction.** ST cells stably expressing shRNAs infected with TGEV at 200 TCID_50_ were incubated with Hoechst 33342 (10 ng/ml) and PI (10 ng/ml) at 30 h after infection. The experiment was performed in triplicate and repeated for three times.

To further investigate the effect of shRNAs on protecting ST cells against TGEV-induced destruction, the cytopathic effect (CPE) and apoptosis of cells stably expressing shRNAs were examined by fluorescence microscopy. The apoptotic cells were characterized by condensed nuclei, and were colored blue by fluorescent dye Hoechst 33342, whereas the CPE in cells that changed from cell fusion to lysis, and thereby formed large bodies, were stained red by propidium iodide (PI). As shown in Figure 7, the negative control pGPU6-GFP/NC had no apparent inhibitory effect on TGEV-induced CPE and apoptosis, because many cells formed large bodies and showed condensed nuclei. In contrast, cells expressing pGPU6-GFP/207 had few condensed nuclei, and almost all cells expressing pGPU6-GFP/207 were capable of maintaining CPE resistance, because there were no apparent PI-stained cells. However, a small area of mild CPE was seen in the cells expressing pGPU6-GFP/241 and pGPU6-GFP/276, indicating that these plasmids were less effective than pGPU6-GFP/207 at protecting ST cells against TGEV-induced destruction.

## Discussion

Although several veterinary coronavirus vaccines are currently available, their efficacy is variable. Among them, the infectious bronchitis virus vaccine is very effective for chickens [23], whereas the canine and porcine vaccines are only partially effective. Furthermore, there is currently no effective antiviral treatment against these virus infections [24]; therefore, rapid and potent anti-TGEV therapeutic agents are urgently needed. RNAi technology provides an important methodology for rational drug design and gene therapy for many viral diseases, which has proven to be a potent tool for host protection against viral infection, suppression of viral genome transcription, and blocking viral replication [25,26]. RNAi can be introduced into the cells using two different approaches. The first is chemically synthesized siRNAs. Cells transfected with chemically synthesized siRNAs can achieve rapid and effective silencing of a target gene, but the effects are transient. Second, shRNAs, which can be cleaved by Dicer to produce siRNAs in the host cell, can circumvent the disadvantages of chemically synthesized siRNAs by using stably transfected plasmids or virus vectors [27-29]. The present study demonstrated the use of RNAi against TGEV via shRNA-expressing plasmid vector pGPU6-GFP, which significantly reduced viral genomic RNA replication and protected ST cells from TGEV destruction, by targeting ORF 7 gene.

RNAi is highly sequence-specific and requires 100% identity between the target and targeting sequences to achieve virus clearance from cell culture [30-32]. The application of shRNAs targeting the conserved region of the gene is one way to overcome the lack of knowledge of the target sequence. To ensure that shRNAs can be used for a wide range of virus strains, we evaluated the cross-inhibitory capabilities of these shRNAs by conducting multiple alignments of TGEV ORF 7 gene sequences, based on the available sequences in GenBank. Our results showed that pGPU6-GFP/207, pGPU6-GFP/241and and pGPU6-GFP/276 could cover 95% (19/20), 75% (15/20) and 75% (15/20) of TGEV strains, respectively (data not shown). Interestingly, we have also conducted multiple alignments of the sequences among TGEV, feline coronavirus virus and canine coronavirus, and have shown that they are also conservative. This conservative characteristic of the sequences between the three viruses means that they have greater potential application for the treatment of the diseases caused by these viruses, which also indicates that further studies are necessary to search for the cross-inhibitory effects in a range of TGEV strains and other coronaviruses.

In our study, two strategies were used to detect the inhibitory effect of shRNAs on both the ORF 7 gene and TGEV genome. The first approach was that cells were infected with TGEV after being transfected with shRNAs, in which the shRNAs were used as preventive substances. As shown in Figures 1 and 2, there was a marked decrease in both the ORF 7 and N genes, which could be amplified simultaneously from all the same regions within the TGEV genome RNA and sub-genomic mRNAs (except the shortest one). The reduction showed that the viral genome RNA and its transcripts were decreased significantly. A similar suppression pattern was observed for virus titers. In the second strategy, shRNAs of ORF 7 gene were used as therapeutic agents and transfected after the cells were infected with TGEV. Almost all the cells maintained their normal morphology before they were collected for real-time RT-PCR. The ORF 7 and N genes were reduced significantly when the shRNAs were transfected at 4 h after TGEV infection (Figures 1 and 2). More experiments were carried out at 8 and 12 h after TGEV infection, but the results were not as remarkable as those after 4 h (data not shown), which indicates that shRNAs affect the initial stage of TGEV infection.

In the study by Ortego et al., recombinant TGEV with ORF 7 gene deletion replicated in cell culture with similar efficiency to the wild-type virus, and stably maintained the modifications introduced into the genome [22]. This observation seems to contrast with the results of the present study, which provides evidence that RNAi of the ORF 7 gene could lead to decreased virus replication. There are three possible reasons for this. First, the genome of parental TGEV could be degraded directly because it consists of a positive-sense ssRNA. Second, all the other sub-genomic mRNAs could be degraded as the ORF 7 gene is included in all the sub-genomic mRNAs. As shown in Figures 2 and 4, N gene was significant decreased in the shRNA expressing cells. Besides, the 3′ end of the TGEV genome gene, the downstream gene of ORF 7 gene, could be degraded with the ORF 7 gene according to the characteristics of RNAi, and its degradation could delay the viral replication, because it has been reported that it could interact with host cell proteins and have a great influence on the replication of TGEV [21]. In the study by Cruz et al., it was reported that TGEV without ORF 7 enhanced virulence in infected piglets [14]. We speculate that this phenomenon won’t happen on the RNAi treated pigs, because the RNAi method is different from the reverse genetics technique. By the RNAi used in this study, the other viral sub-genomic mRNAs were degraded (Figure 2 and 4). While the reverse genetics deletion will only affect the ORF 7 gene itself. Nevertheless, whether the technology could be used in vivo still needs more explored.

To elucidate the protection of shRNAs against TGEV in more detail, ST cells stably expressing the shRNAs were established. TGEV replication in these cells was observed through detection of virus titers and real-time quantitative RT-PCR. Cells expressing pGPU6-GFP/207 showed 99% inhibition in the viral genome, whereas pGPU6-GFP/241 showed up to 89% and pGPU6-GFP/276 85% inhibition (Figure 4). TGEV destroys ST cells in two ways, by apoptosis and necrosis [33,34]. Therefore, CPE and apoptosis were analyzed. Examination of TGEV-induced CPE indicated that ST cells were very sensitive to TGEV infection. Cells expressing pGPU6-GFP/NC formed large bodies and were stained red by PI (Figure 7), whereas cells expressing pGPU6-GFP/207 were not stained red, indicating that the cell membrane was intact. Compared with the condensed, irregular nuclei of pGPU6-GFP/NC-expressing cells, cells expressing pGPU6-GFP/207 were completely protected from apoptosis and the nuclei showed normal morphology. The sequence-specific shRNAs, especially pGPU6-GFP/207, exhibited potent ability to protect ST cells from TGEV-induced destruction. Our present results indicate a close relationship between inhibition of TGEV replication and cell viability by shRNAs. Our data also suggest that siRNA targeting ORF 7 gene can elicit viral RNA from infected cells and potentially offers an efficient therapeutic option for TGEV infection.

## Conclusions

Taken together, our results indicate that plasmid-transcribed shRNAs targeting the ORF 7 gene in the TGEV genome can inhibit expression of viral target genes and viral replication in ST cells. This method merits further investigation in animal studies to define its therapeutic potential, and whether the technology could be used in vivo for anti-TGEV therapy is still under investigation.

## Methods

### Cells and virus

ST cells were cultured in high-glucose Dulbecco’s modified Eagle’s medium (DMEM; GIBCO, UK) containing 10% heat-inactivated fetal calf serum (HyClone, China) and antibiotics (100 μg/ml streptomycin and 100 U/ml penicillin); the culture medium was replaced every 3 days. TGEV H16 strain was obtained from the National Control Institute of Veterinary Bioproducts and Pharmaceuticals (Beijing, China) and propagated in ST cells.

### shRNA sequence selection and plasmid construction

Sequences from the ORF 7 gene of TGEV H16 strain (GenBank accession no: FJ755618) were designed based on the website siRNA designing tools (http://www.ambion.com/techlib/misc/siRNA_finder.html and https://rnaidesigner.invitrogen.com/rnaiexpress/). The sequences were analyzed by BLAST to ensure that they did not have significant nucleotide sequence homology with the swine genome, but shared 100% identity with the other published sequences of TGEV strains, from which four theoretically effective sequences at nucleotide positions 28187–28207 (ORF 7–207), 28218–28238 (ORF 7–238), 28221–28241 (ORF 7–241) and 28256–28276 (ORF 7–276) were selected. A nonspecific sequence (NC) was also scanned by BLAST analysis and served as a negative control. These five sequences are listed in Table 1. All the sequences were arranged in the following alignment: *Bbs*І + Sense + Loop + Antisense + Termination signal + *Bam*HІ and cloned into the pGPU6-GFP vector to make shRNA expressing plasmids: pGPU6-GFP/207, pGPU6-GFP/238, pGPU6-GFP/241, pGPU6-GFP/276 and pGPU6-GFP/NC.

**Table 1:** Inserted sequences in shRNA-expressing plasmids

**Plasmids**	**Inserts**
pGPU6-GFP/207	5’-CACCGGAGTTTAGCAGAAACCAGATTTCAAGAG AATCTGGTTTCTGCTAAACTCCCTAG-3’
pGPU6-GFP/238	5’-CACCGGTGCTTCGAGTAATCTTTCTTTCAAGAGA AGAAAGATTACTCGAAGCACCCTAG-3
pGPU6-GFP/241	5’-CACCGCTTCGAGTAATCTTTCTAGTTTCAAGAGA ACTAGAAAGATTACTCGAAGCCTAG-3’
pGPU6-GFP/276	5’-CACCGCTGCTACAGATTGTTAGTCATTCAAGAGA TGACTAACAATCTGTAGCAGCCTAG-3’
pGPU6-GFP/NC	5’-CACCGTTCTCCGAACGTGTCACGCTTTCAAGAGA AGCGTGACACGTTCGGAGAACCTAG-3’

### TGEV infection of transfected ST cells

Twenty-four hours before being transfected, ST cells were seeded into six-well dishes in high-glucose DMEM + 10% fetal bovine serum (FBS) without antibiotics. When they reached 60–70% confluence, cells were transfected with 4 μg/well pGPU6-GFP/207, pGPU6-GFP/238, pGPU6-GFP/241, pGPU6-GFP/276 and pGPU6-GFP/NC by Lipofectamine 2000 (Invitrogen, Carlsbad, CA, USA) according to the manufacturer’s recommendations. After being incubated at 37°C for 4 h, the transfection complex was removed and the medium was replaced by high-glucose DMEM with 5% FBS. The cells were inoculated with TGEV at 200 TCID_50_ 24 h later. The plasmids contained the GFP gene sequence. Therefore, GFP was expressed allowing a good discrimination between transfected and non-transfected cells. The transfection efficiency was determined by monitoring the percentage of GFP-expressing cells within the live cell population, and it showed that nearly 80% of the transfected cells were positive. CPE was evaluated and the cell images were captured under an inverted fluorescence/phase-contrast microscopy (Nikon, Japan) at different time points post-infection. The cell cultures were collected for a real-time quantitative RT-PCR analysis at 40 h post-infection.

### shRNA transfection in TGEV-infected ST cells

One-day-old ST cells (60–70% confluence) were infected with TGEV at 200 TCID_50_, and 4 h later, the transfection complex of the shRNA-expressing plasmids (4 μg/well) was added to each well and incubated for 4 h. The medium was replaced by high-glucose DMEM + 5% FBS, and the cells were further incubated at 37°C in a 5% CO_2_ atmosphere. Cell images were captured and the cell cultures were collected for real-time quantitative RT-PCR analysis at 40 h post-infection.

### Total RNA extraction and real-time quantitative RT-PCR

To investigate the effect of the plasmid-transcribed shRNAs on TGEV ORF 7 gene, TGEV-infected ST cells were collected 40 h after viral infection. Total RNA was isolated by Trizol phenol–chloroform extraction (Invitrogen) and ethanol precipitation, following the manufacturer’s recommendations. A real-time quantitative RT-PCR was conducted by using a SYBR ExScript™ RT-PCR Kit (Takara Bio, Dalian, China), according to manufacturer’s instructions. The following two pairs of primers: forward, 5′-TTGCTCGTCCTCCTCCATGC-3′ and reverse, 5′-CCACTTTTAGTAATCTGGTTTCTGC-3′ for TGEV ORF 7 gene; forward, 5′-CGTCCACCGCAAATGCTTC-3′ and reverse, 5′-AACCGACTGCTGTCACCTTCAC-3′ for β-actin gene. PCR was performed in an iQ5 Real-Time PCR Detection System (Bio-Rad, USA). Following a denaturation step at 95°C for 10 s, 42 cycles of amplification were performed at 95°C for 5 s, 58°C for 10 s, and 72°C for 15 s. Data were analyzed according to the Ct method, where the amount of RNA in samples normalized to β-actin and the tests was determined in triplicate.

To assess the influence of shRNAs on TGEV replication, the N gene of TGEV was used as a standard for the TGEV genome. Another pair of primers was synthesized for quantification of the TGEV genome in real-time RT-PCR: forward, 5′- GGAAGATGGCGACCAGATAG-3′ and reverse, 5′- CCACTTCTGATGGACGAGCA-3′. Total RNA of culture supernatants was isolated as described above, and a real-time quantitative RT-PCR was conducted using a SYBR ExScript™ RT-PCR Kit (Takara Bio), according to the manufacturer’s instructions. Real-time RT-PCR was performed and the procedure was similar to that described above, expect that the reaction temperature for annealing was 60°C. Data were analyzed according to the Ct method, and the tests were performed in triplicate.

### Virus titration

TGEV cultures in siRNA-transfected cells were collected 48 h after viral infection. After three freeze–thaw cycles, the cultures were serially diluted 10-fold from 10^–1^ to 10^–10^, and added to ST cells at 50–60% confluence in 96-well plates. Each dilution was added to four wells. After 3 days of infection, the TCID_50_ was calculated by the Reed–Muench method.

### Establishment of ST cell lines stably expressing shRNAs

The pGPU6-GFP vector carries the neomycin resistance gene; therefore, ST cells stably expressing shRNAs were selected using G418. GFP in the plasmids was used as a reporter during the selection efficiency analysis. The ST cells were seeded into six-well plates 24 h before being transfected (up to 60–70% confluence). Cells were transfected with pGPU6-GFP/207, pGPU6-GFP/241, pGPU6-GFP/276 and pGPU6-GFP/NC by Lipofectamine 2000 as described before, and propagated in selection medium containing 1500 μg/ml G418 until 90% of the surviving cells stably expressed GFP.

### MTS assay

ST cells stably expressing pGPU6-GFP/207, pGPU6-GFP/241, pGPU6-GFP/276 and pGPU6-GFP/NC were seeded in 96-well plates at 50–60% confluence and challenged with TGEV at 200 TCID_50_. Cell viability was assessed by adding 20 μl/well of MTS (Promega, USA) to cell cultures, according to the manufacturer’s instructions, at 36 h after viral infection. After incubation with MTS for 4 h, light absorbance of each well was measured at 492 nm. Each measurement was performed six times, and the experiment was repeated three times.

### Fluorescence microscopy

ST cells stably expressing shRNAs were seeded in six-well tissue culture plates at 50–60% confluence and challenged with TGEV at 200 TCID_50_. Thirty hours after infection, the cells were washed with Hank’s balanced salt solution (HBSS) and incubated with Hoechst 33342 (10 ng/ml) at 37°C for 15 min, and then washed three times with HBSS. Cell were then incubated with PI (10 ng/ml) at 37°C for 15 min and washed with DMEM without serum. Images were viewed by fluorescence microscopy (Nikon, Japan).

## Abbreviations

TGE: Transmissible gastroenteritis; TGEV: Transmissible gastroenteritis virus; ORF 7: Open reading frame 7; RdRp: RNA-dependent RNA polymerase; RNAi: RNA interference; shRNAs: short hairpin RNAs; ST: Swine testicular; CPE: Cytopathic effect; GFP: Green fluorescence protein; TCID_50_: 50% Tissue culture infective dose; Ct: Comparative threshold; PI: Propidium Iodide; DMEM: Dulbecco’s modified eagle medium; SD: Standard deviation; FBS: Fetal bovine serum; HBSS: Hank’s balanced salt solution.

## Competing interests

The authors declare that they have no competing interests.

## Authors’ contributions

Lei He took part in all the experiments, and wrote the manuscript. Yan-ming Zhang designed all the experiments. Ling-juan Dong and Min Cheng participated in plasmid construction, cell transfection and confocal microscopy. Jing Wang made a major contribution to the MTS assay. Gang Wang and Qing-hai Tang carried out the cell culture and real-time RT-PCR. All authors read and approved the final manuscript.

## References

[B1] Jones T Pritchard G Paton D Transmissible gastroenteritis of pigs Vet Rec 1997 141 427 428 9364718

[B2] Wesley RD Woods RD Cheung AK Genetic basis for the pathogenesis of transmissible gastroenteritis virus J Virol 1990 64 4761 4766 2168963 10.1128/jvi.64.10.4761-4766.1990 PMC247963

[B3] Schwegmann-Wessels C Zimmer G Schroder B Breves G Herrler G Binding of transmissible gastroenteritis coronavirus to brush border membrane sialoglycoproteins J Virol 2003 77 11846 11848 10.1128/JVI.77.21.11846-11848.2003 14557669 PMC229351

[B4] Holmgren J Czerkinsky C Eriksson K Mharandi A Mucosal immunisation and adjuvants: a brief overview of recent advances and challenges Vaccine 2003 21 Suppl 2 S89 S95 12763689 10.1016/s0264-410x(03)00206-8

[B5] Wesley RD Lager KM Increased litter survival rates, reduced clinical illness and better lactogenic immunity against TGEV in gilts that were primed as neonates with porcine respiratory coronavirus (PRCV) Vet Microbiol 2003 95 175 186 10.1016/S0378-1135(03)00150-0 12935745 PMC7117301

[B6] Brim TA VanCott JL Lunney JK Saif LJ Cellular immune responses of pigs after primary inoculation with porcine respiratory coronavirus or transmissible gastroenteritis virus and challenge with transmissible gastroenteritis virus Vet Immunol Immunopathol 1995 48 35 54 10.1016/0165-2427(94)05416-P 8533315 PMC7119789

[B7] Ding SW Li H Lu R Li F Li WX RNA silencing: a conserved antiviral immunity of plants and animals Virus Res 2004 102 109 115 10.1016/j.virusres.2004.01.021 15068886

[B8] Fire A Xu S Montgomery MK Kostas SA Driver SE Mello CC Potent and specific genetic interference by double-stranded RNA in Caenorhabditis elegans Nature 1998 391 806 811 10.1038/35888 9486653

[B9] Porntrakulpipat S Supankong S Chatchawanchonteera A Pakdee P RNA interference targeting nucleocapsid protein (C) inhibits classical swine fever virus replication in SK-6 cells Vet Microbiol 2010 142 41 44 10.1016/j.vetmic.2009.09.041 19850420

[B10] Chen W Liu M Jiao Y Yan W Wei X Chen J Fei L Liu Y Zuo X Yang F Adenovirus-mediated RNA interference against foot-and-mouth disease virus infection both in vitro and in vivo J Virol 2006 80 3559 3566 10.1128/JVI.80.7.3559-3566.2006 16537624 PMC1440392

[B11] Xu X Guo H Xiao C Zha Y Shi Z Xia X Tu C In vitro inhibition of classical swine fever virus replication by siRNAs targeting Npro and NS5B genes Antiviral Res 2008 78 188 193 10.1016/j.antiviral.2007.12.012 18262291

[B12] Zhou JF Hua XG Cui L Zhu JG Miao DN Zou Y He XZ Su WG Effective inhibition of porcine transmissible gastroenteritis virus replication in ST cells by shRNAs targeting RNA-dependent RNA polymerase gene Antiviral Res 2007 74 36 42 10.1016/j.antiviral.2006.12.007 17287033 PMC7114347

[B13] Zhou J Huang F Hua X Cui L Zhang W Shen Y Yan Y Chen P Ding D Mou J Inhibition of porcine transmissible gastroenteritis virus (TGEV) replication in mini-pigs by shRNA Virus Res 2010 149 51 55 10.1016/j.virusres.2009.12.012 20080134 PMC7126616

[B14] Cruz JL Sola I Becares M Alberca B Plana J Enjuanes L Zuniga S Coronavirus gene 7 counteracts host defenses and modulates virus virulence PLoS Pathog 2011 7 e1002090 10.1371/journal.ppat.1002090 21695242 PMC3111541

[B15] Enjuanes L Almazan F Sola I Zuniga S Biochemical aspects of coronavirus replication and virus-host interaction Annu Rev Microbiol 2006 60 211 230 10.1146/annurev.micro.60.080805.142157 16712436

[B16] Bernard S Laude H Site-specific alteration of transmissible gastroenteritis virus spike protein results in markedly reduced pathogenicity J Gen Virol 1995 76 Pt 9 2235 2241 7561760 10.1099/0022-1317-76-9-2235

[B17] Alonso S Izeta A Sola I Enjuanes L Transcription regulatory sequences and mRNA expression levels in the coronavirus transmissible gastroenteritis virus J Virol 2002 76 1293 1308 10.1128/JVI.76.3.1293-1308.2002 11773405 PMC135778

[B18] Yin JC Ren XF Li YJ Molecular cloning and phylogenetic analysis of ORF7 region of chinese isolate TH-98 from transmissible gastroenteritis virus Virus Genes 2005 30 395 401 10.1007/s11262-004-6783-y 15830158 PMC7089185

[B19] Tung FY Abraham S Sethna M Hung SL Sethna P Hogue BG Brian DA The 9-kDa hydrophobic protein encoded at the 3' end of the porcine transmissible gastroenteritis coronavirus genome is membrane-associated Virology 1992 186 676 683 10.1016/0042-6822(92)90034-M 1310191 PMC7130826

[B20] Park JH Han JH Kwon HM Sequence analysis of the ORF 7 region of transmissible gastroenteritis viruses isolated in Korea Virus Genes 2008 36 71 78 10.1007/s11262-007-0191-z 18172751

[B21] Galan C Sola I Nogales A Thomas B Akoulitchev A Enjuanes L Almazan F Host cell proteins interacting with the 3' end of TGEV coronavirus genome influence virus replication Virology 2009 391 304 314 10.1016/j.virol.2009.06.006 19580983 PMC7118768

[B22] Ortego J Sola I Almazan F Ceriani JE Riquelme C Balasch M Plana J Enjuanes L Transmissible gastroenteritis coronavirus gene 7 is not essential but influences in vivo virus replication and virulence Virology 2003 308 13 22 10.1016/S0042-6822(02)00096-X 12706086 PMC7126239

[B23] Ladman BS Pope CR Ziegler AF Swieczkowski T Callahan CJ Davison S Gelb J Jr Protection of chickens after live and inactivated virus vaccination against challenge with nephropathogenic infectious bronchitis virus PA/Wolgemuth/98 Avian Dis 2002 46 938 944 10.1637/0005-2086(2002)046[0938:POCALA]2.0.CO;2 12495055

[B24] Pratelli A Tinelli A Decaro N Martella V Camero M Tempesta M Martini M Carmichael LE Buonavoglia C Safety and efficacy of a modified-live canine coronavirus vaccine in dogs Vet Microbiol 2004 99 43 49 10.1016/j.vetmic.2003.07.009 15019110 PMC7117189

[B25] Wu K Mu Y Hu J Lu L Zhang X Yang Y Li Y Liu F Song D Zhu Y Wu J Simultaneously inhibition of HIV and HBV replication through a dual small interfering RNA expression system Antiviral Res 2007 74 142 149 10.1016/j.antiviral.2006.11.004 17173982 PMC7114121

[B26] Li J Guo H Shi Z Tu C In vitro inhibition of CSFV replication by retroviral vector-mediated RNA interference J Virol Methods 2010 169 316 321 10.1016/j.jviromet.2010.07.036 20691206 PMC7112837

[B27] Peng S York JP Zhang P A transgenic approach for RNA interference-based genetic screening in mice Proc Natl Acad Sci USA 2006 103 2252 2256 10.1073/pnas.0511034103 16461920 PMC1413752

[B28] Brummelkamp TR Bernards R Agami R A system for stable expression of short interfering RNAs in mammalian cells Science 2002 296 550 553 10.1126/science.1068999 11910072

[B29] Berns K Hijmans EM Mullenders J Brummelkamp TR Velds A Heimerikx M Kerkhoven RM Madiredjo M Nijkamp W Weigelt B A large-scale RNAi screen in human cells identifies new components of the p53 pathway Nature 2004 428 431 437 10.1038/nature02371 15042092

[B30] Gitlin L Karelsky S Andino R Short interfering RNA confers intracellular antiviral immunity in human cells Nature 2002 418 430 434 10.1038/nature00873 12087357

[B31] Lambeth LS Zhao Y Smith LP Kgosana L Nair V Targeting Marek's disease virus by RNA interference delivered from a herpesvirus vaccine Vaccine 2009 27 298 306 10.1016/j.vaccine.2008.10.023 18977264

[B32] Randall G Grakoui A Rice CM Clearance of replicating hepatitis C virus replicon RNAs in cell culture by small interfering RNAs Proc Natl Acad Sci USA 2003 100 235 240 10.1073/pnas.0235524100 12518066 PMC140937

[B33] Kim B Kim O Tai JH Chae C Transmissible gastroenteritis virus induces apoptosis in swine testicular cell lines but not in intestinal enterocytes J Comp Pathol 2000 123 64 66 10.1053/jcpa.2000.0386 10906258

[B34] Eleouet JF Slee EA Saurini F Castagne N Poncet D Garrido C Solary E Martin SJ The viral nucleocapsid protein of transmissible gastroenteritis coronavirus (TGEV) is cleaved by caspase-6 and −7 during TGEV-induced apoptosis J Virol 2000 74 3975 3983 10.1128/JVI.74.9.3975-3983.2000 10756009 PMC111911

